# Measuring Food and Water Security in an Aboriginal Community in Regional Australia

**DOI:** 10.1111/ajr.13214

**Published:** 2025-01-12

**Authors:** Loretta Weatherall, Alinta Trindall, Trish Tonkin, Joseph Alvin Santos, Dori Patay, Ruth McCausland, Wendy Spencer, Greg Leslie, Eileen Baldry, Keziah Bennett‐Brook, Julieann Coombes, Tamara Mackean, Janani Shanthosh, Ty Madden, Bruce Moore, Ann‐Marie Deane, Niall Earle, Christine Corby GDip, Melissa Nathan, Sera L. Young, Emalie Rosewarne, Jacqui Webster

**Affiliations:** ^1^ Walgett Aboriginal Medical Service Limited Walgett New South Wales Australia; ^2^ The George Institute for Global Health University of New South Wales Sydney New South Wales Australia; ^3^ Yuwaya Ngarra‐Li Partnership, University of New South Wales Sydney New South Wales Australia; ^4^ Dharriwaa Elders Group Walgett New South Wales Australia; ^5^ Global Water Institute University of New South Wales Sydney New South Wales Australia; ^6^ Faculty of law and Justice University of New South Wales Sydney New South Wales Australia; ^7^ College of Medicine and Public Health Flinders University Adelaide South Australia Australia; ^8^ Anthropology and Global Health Studies Northwestern University Evanston Illinois USA

**Keywords:** Aboriginal and Torres Strait Islander, First Nations Health, food security, remote and regional communities, water security

## Abstract

**Objective:**

To measure current levels and experiences of food and water security in Walgett to guide a community‐led program and to provide a baseline measure.

**Design:**

A community‐led cross‐sectional survey conducted in April 2022 by trained local researchers.

**Setting:**

Walgett, a regional town in NSW, Australia.

**Participants:**

A total of 251 Aboriginal adults.

**Main Outcome Measured:**

Food and water security levels and experiences were measured using the Household Food Insecurity Access Scale (HFIAS) and Household Water InSecurity Experiences (HWISE) Scale. The relationship between food and water insecurity was determined through linear regression analysis.

**Results:**

Almost half of the respondents experienced food insecurity (46%) or water insecurity (44%) in the last 12 months. Most participants attributed food insecurity to difficulties with food affordability (71%) and availability (63%). More than four in five participants reported relying on purchased or donated bottled water due to main water source interruption (83%) or quality concerns (86%). Water insecurity was associated with food insecurity; HFIAS score increased by 0.43 points for every point higher on the HWISE scale.

**Conclusions:**

This study is the first to measure levels and experiences of food and water security in an Aboriginal community in Australia using validated tools. The results highlight the interconnectedness of food and water insecurity and provide evidence of levels far higher than Australian national level estimates and comparable to low‐ and middle‐income countries. A holistic government response alongside community‐led efforts are needed to increasefood and water security to improve health and well‐being in remote Aboriginal communities.

**Summary**

- What is already known on this subject?
  ○ There are no measures of food and water security in Walgett, and other Aboriginal communities in Australia, using culturally adapted validated tools.
  ○ In Walgett, community knowledge and experiences of severe water and food insecurity issues have not been heard or addressed by Government.
- What this study adds?
  ○ This study provides evidence that the level of food and water security in Walgett, a remote Aboriginal community, is on par with several low‐/middle‐income countries.
  ○ It highlights the interconnectedness of food and water security, which should underpin further action.
  ○ We need to go beyond a single‐item question in national surveys and use culturally adapted validated tools to measure food and water security across the nation.

## 1 Introduction

For Aboriginal and Torres Strait Islander peoples, food and water security is a combined, holistic concept based on an ongoing spiritual, physical and cultural connection between people and Country [1]. However, colonisation fractured Aboriginal and Torres Strait Islanders peoples' connection to Country, including traditional food and water systems and diets by cutting off natural food and water resources [2]. This severing of connection to Country and culture and the introduction of Western diets have been key drivers of food and water insecurity [1, 2]. Food and water security are requisites of a healthy diet and the absence of either will have an adverse impact on nutrition, resulting in increased rates of diet‐related chronic diseases [3, 4]. Poor nutrition is the primary contributor to the increasing health gap between Aboriginal and Torres Strait Islander communities and non‐Indigenous communities in Australia [5]. The Close the Gap Campaign aims to close the health and life expectancy gap between Aboriginal and Torres Strait Islander peoples and non‐Indigenous Australians within a generation, including recognising the human right to an adequate standard of living, including food and water [6].

Food and water security in regional and remote Australian towns, however, have been further jeopardised by bushfires, floods and drought brought on by climate change. In particular, extended drought coupled with increased floodplain harvesting has reduced run‐off and surface water flows, impacting drinking water quality and local supplies of freshwater and fish [7]. In regional and remote communities, food prices are reportedly around 30% higher [8], and income levels are typically lower, making healthy diets less affordable [9]. Existing food and water security challenges in regional Australia have been further exacerbated by the recent COVID‐19 pandemic and the war in Ukraine, which have impacted global, national and regional food supplies and prices [10, 11].

In the absence of effective government action, communities are establishing their own programs to improve food and water security. The Dharriwaa Elders Group (DEG) and Walgett Aboriginal Medical Service (WAMS) [7] have worked together for decades to improve outcomes for the Aboriginal community in Walgett, a remote town in New South Wales (Australia), which includes people from the Gamilaraay, Ngayiimbaa, Wayilwan and Yuwaalaraay nations. Following a series of threats to food and water security in Walgett, including prolonged droughts, the drying of the rivers resulting in loss of native food sources, COVID‐19 and the local supermarket burning down, the Aboriginal community prioritised the need to establish resilient food and water systems [2].

In April 2019, the Yuwaya Ngarra‐li partnership between DEG and the University of New South Wales (UNSW) convened a forum to bring the community together to discuss positive strategies and evidence‐based solutions [2]. This led to the establishment of the Walgett Food and Water for Life Project: a community‐led initiative developing and implementing innovative solutions to food and water security, including public filtered drinking water kiosks, healthier supermarket options, more resources for community gardens and advocating for systemic changes to ensure a sustainable, secure, local supply of affordable, nutritious food and safe drinking water [12]. The objective of this study was to measurecurrent levels of food and water security in Walgett, and understand how these conditions are experienced by Aboriginal adults within the community. This is both to guide the program of solutions and to serve as a baseline from which to monitor subsequent change.

## 2 Materials and Methods

### 2.1 Terminology

This study is placed in remote New South Wales (Australia) with a substantial Aboriginal population; hence, we refer mainly to Aboriginal people throughout this paper. References to Torres Strait Islander people will be specifically stated where relevant as we recognise that Aboriginal and Torres Strait Islander cultures are very different, with their own unique histories, beliefs and values.

### 2.2 Community Leadership

This study is part of an ongoing partnership between academics and Aboriginal community‐controlled organisations. Aboriginal community researchers were involved in the study design as part of the original grant writing team and through development of study protocols, training and data gathering, analysis of data and writing of the paper through writing workshops to develop early drafts and providing input on subsequent drafts. This work was done in accordance with the STROBE guidelines for observational studies (Table S1).

An intensive 2‐day training and planning session took place in Walgett immediately prior to the survey and the survey team reported back daily to share lessons and ensure the tools were being used consistently.

### 2.3 Study Setting

Walgett is a remote community in NSW with almost half its population of approximately 2200 people identifying as Aboriginal [13].^1^ Aboriginal people have long‐held cultural and spiritual connections to Country and to the Namoi and Barwon rivers of the area. Like many remote Australian communities, Walgett faces severe food and water security challenges [2]. Between 2018 and 2020, the local river water supply steadily declined due to drought and over‐extraction upstream, resulting in the extended and exclusive use of groundwater sourced from the Great Artesian Basin as the town water supply [7]. Since then, the community has reported various issues, including supply interruptions and poor aesthetic quality, particularly unpalatability [7]. Walgett groundwater has been found to have sodium levels almost twice the Australian Drinking Water Guidelines (ADWG) for palatability and 15 times the levels recommended for people with high blood pressure [2, 7]. This is unacceptable since hypertension is already 30% higher in Aboriginal and Torres Strait Islander communities than the general Australian population [14]. Food security was threatened when the only local supermarket burnt down twice in the last decade (2013 and 2019) meaning the nearest main food source was over 80 km away [15].

### 2.4 Research Design and Data Collection

Cross‐sectional surveys were conducted among Aboriginal people, or those living with/caring for Aboriginal people, aged ≥ 18 years, living in Walgett town or nearby Gingie and Namoi villages, in April 2022. The survey was conducted by Aboriginal staff from DEG and WAMS, with support from the George Institute for Global Health, which included training on data collection.

The survey tools were based on existing global tools [16, 17], but following extensive discussion with the local Aboriginal organisations, four supplementary questions were added to the water security tool and a free‐text question was added after both the food and the water security sections. The questions were pre‐tested with community members through practice interviews, and small changes were made to the language to ensure cultural appropriateness. Data on socio‐demographic factors and food and water security experiences over the last 12 months were collected (See Table S2). Food insecurity questions included nine items from the Household Food Insecurity Access Scale (HFIAS) [18] and three supplementary questions determined by the community during testing. Water insecurity questions included 12 items from the Household Water InSecurity Experiences (HWISE) Scale [17] plus seven additional questions identified during testing.

Survey administrators were stationed outside the supermarket to permit convenience sampling. The intention was to recruit an equal number of participants in each age and sex band (i.e., male 18–44 years, male ≥ 45 years, female 18–44 years and female ≥ 45 years). Potentially eligible participants were approached and provided details about the study objectives, risks and benefits to permit informed written consent. Surveys were verbally administered face‐to‐face in nearby WAMS tents or DEG offices. Participants were given the option to not answer anyquestions they felt uncomfortable with. On completion of the survey, participants received a $25 AUD gift‐voucher redeemable at local shops.

### 2.5 Data Analysis

Food insecurity scores were calculated by summing the responses to the nine HFIAS items (range 0–27) as per Dekker, de Graff and Marijni's method [19]. Water insecurity scores were calculated by summing the 12 HWISE items (range 0–36). Higher scores indicate a higher level of food or water insecurity, with cut‐offs of ≥ 9 and ≥ 12 for food and water insecurity, respectively [17, 19]. Responses to HFIAS and HWISE questions were coded as 0 for *never*, 1 for *in* 1 or 2 *months*, 2 for *in some months* and 3 for *in most months*. Respondents with missing responses on ≥ 25% of the questions were excluded from the HFIAS and HWISE score calculations to avoid underestimation of the mean scores. Tables S3 and S4 illustrates the mapping of questions from the global survey tools to this survey.

Descriptive statistics were used to summarise participant characteristics, responses to food and water security questions, and the HFIAS and HWISE scores. Differences by sex, age group (18–44 years vs. ≥ 45 years) and location (Walgett town or other) were determined using a chi‐squared test (for categorical variables), an independent samples *t*‐test or Wilcoxon rank sum test (for continuous variables). The relationship between food and water insecurity was determined through linear regression, with the HFIAS score as the dependent variable and the HWISE score as the independent variable, adjusted for sex, age and education level. These variables were selected based on their inclusion in the dataset and clinical importance. Analyses were conducted using Stata IC 16.0 for Windows (StataCorp LP, College Station, TX, USA). Figures were generated using ggplot2 in R [20].

### 2.6 Power and Sample Size

A sample size of 250 was estimated to achieve at least 80% power to detect a 10% difference in the proportion of the sample who were food or water secure, based on a longitudinal design with two repeated measurements, assuming a within‐subject correlation of 0.400 and α = 0.05. This sample size would also achieve at least 80% power to detect at least a 17% difference in proportions between two independent groups (e.g., males versus females).

### 2.7 Ethics Approval

The study was conducted in accordance with the Declaration of Helsinki. Ethics approval was obtained from the Aboriginal Health and Medical Research Council of New South Wales (1781/20) and was ratified by the University of New South Wales Human Research Ethics Committee. All participating adults provided written consent.

## 3 Results

### 3.1 Participant Characteristics

The survey was completed by 251 participants (Table 1). The final sample broadly reflected the recent Australian census demographic distributions in terms of sex (55% female) and age (54% ≥ 45 years) representation.

**TABLE 1:** Socio‐demographic characteristics of participants in the surveys.

Characteristics	Overall (*n* = 251)	Males (*n* = 112)	Females (*n* = 139)
Age, years (mean, SD)	45.7 (15.9)	46 (15.2)	45.5 (16.4)
Age group (*n*, %)			
18–44 years	116 (46)	51 (46)	65 (47)
45 years and up	135 (54)	61 (55)	74 (53)
Aboriginal origin (*n*, %)			
No	8 (3)	1 (1)	7 (5)
Yes	240 (97)	111 (99)	129 (95)
Location (*n*, %)			
Walgett town	203 (81)	88 (79)	115 (83)
Gingie village/Namoi village/out of town Walgett area	46 (18)	22 (20)	24 (17)
Unknown	2 (1)	2 (2)	0
Highest level of education (*n*, %) [1]			
Primary level	25 (10)	11 (10)	14 (10)
Secondary level	185 (75)	91 (82)	94 (69)
Tertiary level	38 (15)	9 (8)	29 (21)
How many people do you usually live with? (*n*, %)			
Live alone	23 (9)	14 (13)	9 (7)
Live with partner	25 (10)	11 (10)	14 (10)
Shared household (2–5 people)	166 (66)	74 (66)	92 (66)
Shared household (> 6 people)	37 (15)	13 (12)	24 (17)
How many people do you usually eat with? (*n*, %)			
Eat alone	23 (9)	13 (12)	10 (7)
Eat with partner	21 (8)	10 (9)	11 (8)
Shared household (2–5 people)	163 (65)	74 (66)	89 (65)
Shared household (6–8 people)	39 (16)	14 (13)	25 (18)
Shared household (> 8 people)	4 (2)	1 (1)	3 (2)
Which of the following best describes your role? (*n*, %) [1]			
Responsible for shopping and cooking most of the time	141 (58)	48 (43)	93 (69)
Responsible for shopping most of the time	6 (2)	3 (3)	3 (2)
Responsible for cooking most of the time	2 (1)	1 (1)	1 (1)
Share responsibility for shopping and cooking	75 (31)	43 (39)	32 (24)
Not responsible for shopping and cooking	21 (9)	16 (14)	5 (4)

### 3.2 Overview of Food and Water Insecurity in Walgett

Thirty per cent of participants experienced both food and water insecurity (Table 2). Almost half of participants (46%) were food insecure (HFIAS > 9) and 44% were water insecure (HWISE ≥ 12). Participants that experienced water insecurity were more likely to experience food insecurity; HFIAS score increased by 0.43 (95% CI 0.35–0.52; *p* < 0.001) per unit increase in the HWISE score. We primarily report on overall group results in this paper, although there were some differences between socio‐demographic subgroups (Tables S5–S8).

**TABLE 2:** Food insecurity (*n* = 251) and water insecurity (*n* = 247) experienced in the Walgett community, overall and by sex, age and location.

	Overall	By sex		by age group		By location^a^	
	(*n* = 251)	Males (*n* = 112)	Females (*n* = 139)	18–44 years (*n* = 116)	45 years and up (*n* = 135)	Walgett town (*n* = 203)	Other areas^b^ (*n* = 46)
HFIAS score (mean, SD)*	8.4 (6.9)	8.2 (6.6)	8.5 (7.1)	7.4 (6.3)	9.3 (7.2)	8.3 (7.0)	9.1 (6.3)
HFIAS score (median, IQR)	7 (2–13)	8 (2–12)	7 (2–14)	6.5 (1.5–11.5)	8 (3–16)	7 (2–13)	9 (4–12)
HFIAS classification (*n*, %)							
< 9	136 (54)	60 (54)	76 (55)	67 (58)	69 (51)	114 (56)	21 (46)
≥ 9	115 (46)	52 (46)	63 (45)	49 (42)	66 (49)	89 (44)	25 (54)
HWISE score (mean, SD)	11.01 (8.7)	10.4 (7.8)	11.50 (9.4)	10.8 (8.7)	11.15 (8.9)	11.51 (8.8)	9.1 (8.4)
HWISE score (median, IQR)	10 (4–16)	9.5 (4–16)	10 (4–17)	9 (4–16)	10 (4–16)	10 (4–17)	7.5 (3–13)
HWISE classification (*n*, %)^c^							
< 12	139 (56)	64 (58)	75 (55)	67 (59)	72 (54)	106 (53)	31 (67)
≥ 12	108 (44)	46 (42)	62 (45)	46 (41)	62 (46)	93 (47)	15 (33)
Both food and water insecure^d^							
No	174 (70)	79 (72)	95 (69)	86 (76)	88 (66)	139 (70)	33 (72)
Yes	73 (30)	31 (28)	42 (31)	27 (24)	46 (34)	60 (30)	13 (28)

^a^ Analysis by location excludes two respondents with unknown location.

^b^ Other areas include Gingie Village, Namoi Village, and out of town Walgett area.

^c^ Four respondents were excluded from the calculation of HWISE score since they had ≥ 3 (or ≥ 25%) missing response on the 12 questions. This was done to avoid underestimation of the HWISE score.

^d^ HFIAS ≥ 9 AND HWISE ≥ 12.

* Significant difference by age group at *p* < 0.05.

#### 3.2.1 Food Insecurity

**FIGURE 1:**
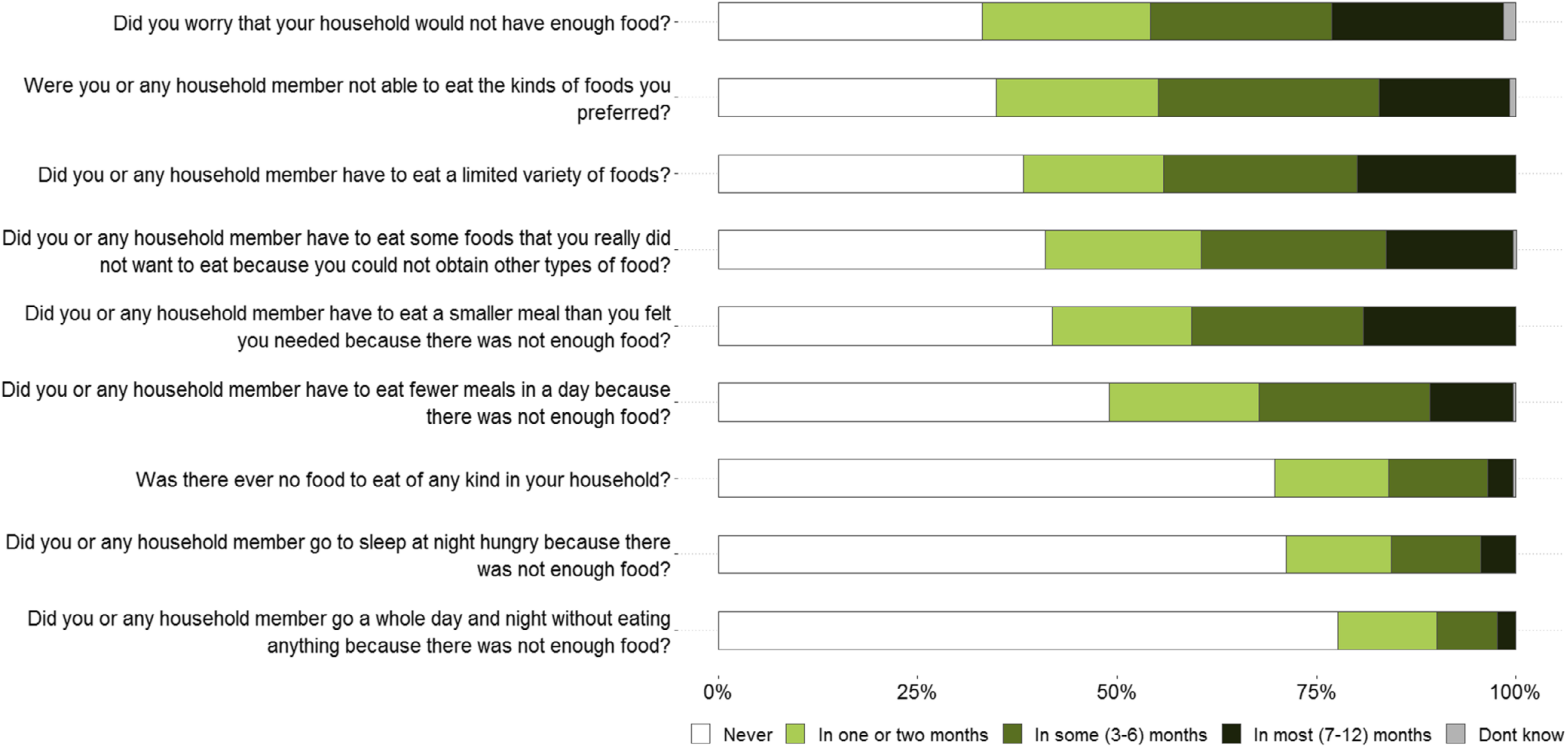
Frequency of food insecurity experienced by participants during the last 12 months using the Household Food Insecurity Access Scale (*n* = 251).

Around two‐thirds of people reported worrying about not having enough food (67%), not being able to eat preferred foods (65%) and eating a limited variety of foods (62%) (Figure 1). Many participants experienced having no food to eat (30%), going to sleep hungry (29%) and going a whole day and night without eating (22%) in at least 1 month in the last 12 months. For around one‐fifth of participants, they worried about not having enough food (22%), eating a limited variety of foods (20%) and eating smaller meals (19%), in most months. There were few differences in HFIAS scores by sex, age and location (Table S5). However, the frequency of females versus males experiencing the three most severe indicators of food insecurity did vary (i.e., having no food to eat of any kind in your household, going to sleep at night hungry because there was not enough food or going a whole day and night without eating because there was not any food).

#### 3.2.2 Other Food Security Issues Raised

When asked whether there was anything else they wanted to add about food, affordability (*n* = 82) was most frequently raised by participants. One participant explained: ‘Prices of food and fresh food are too expensive, especially for low‐income families in a small community like Walgett’ (Female, 23 years). People spoke about high prices impacting their food choices, for example, how they ‘go and buy takeaway because it's cheaper’ (Female, 37 years) and that they ‘can't buy most things [they] would like to eat’ (Female, 44 years). Participants discussed the lack of availability (*n* = 22) and poor quality (*n* = 19) of fresh food in the local supermarket, for example, ‘It is sometimes hard to get fruit and veg and sometimes they are squashed or damaged’ (Female, 61 years). Utilisation (e.g., unsafe food), accessibility of the supermarket (e.g., open hours, distance from home) and low variety (e.g., fresh or tinned vegetables) were other concerns.

#### 3.2.3 Water Insecurity

**FIGURE 2:**
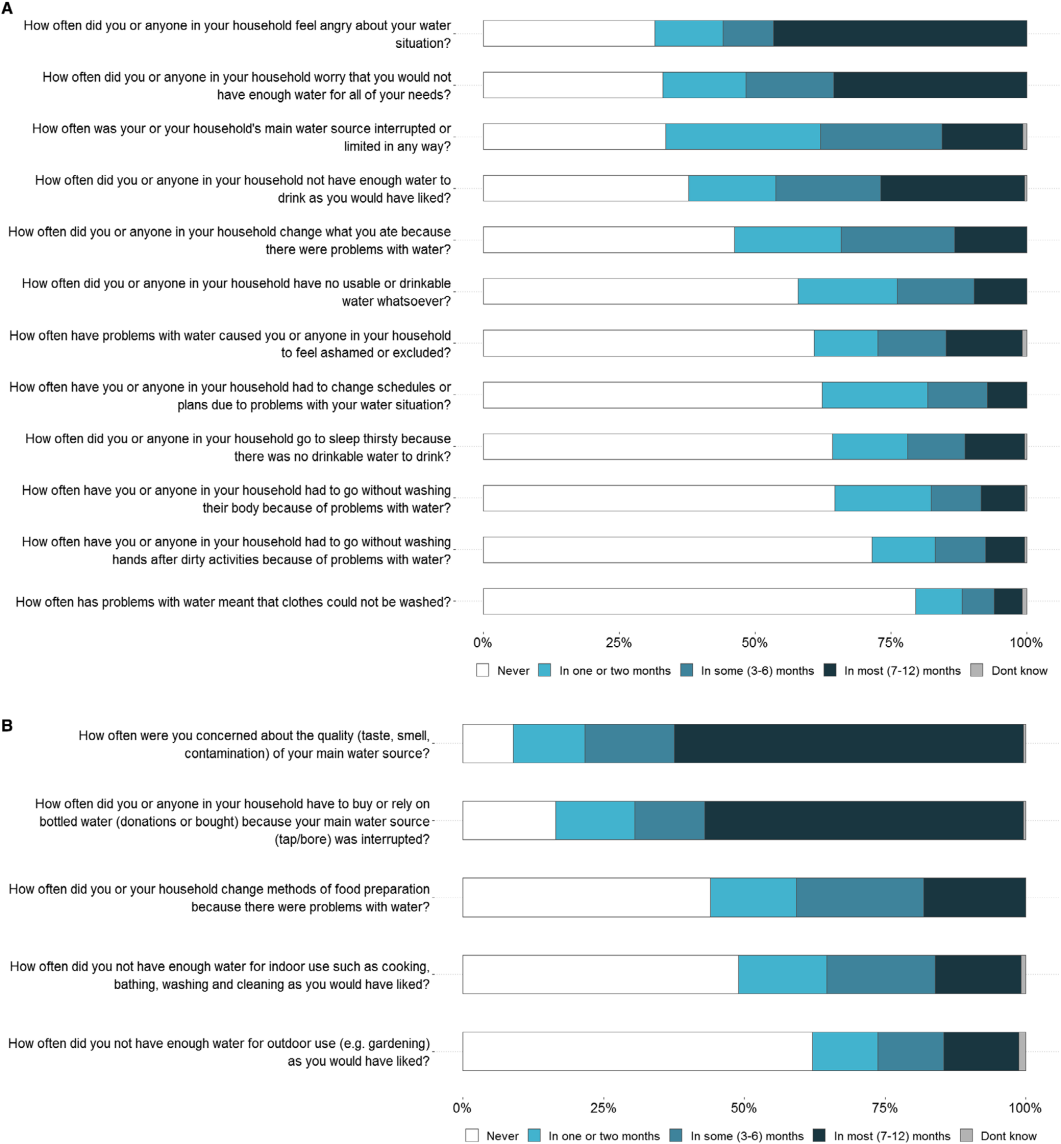
(A) Frequency of water insecurity indicators experienced by participants during the last 12 months using the Household Water InSecurity Experiences Scale (*n* = 247). (B) Frequency of other water insecurity indicators experienced by participants during the last 12 months (*n* = 247).

The most commonly reported water security issues were feeling angry about the water situation (68%), worrying about not having enough water (67%) and having the household's main water supply interrupted or limited (66%) in at least 1 month in the last year (Figure 2). Almost half of the participants reported feeling angry about the water situation (47%) and over one‐quarter experienced not having enough water to drink (27%) in most months in the last year. Many participants reported having no usable or drinkable water whatsoever (42%) and going to sleep thirsty (36%) in at least 1 month in the last year. Around 10% of the participants reported having to change what they ate because of the water, having to go to sleep thirsty, and having the household's main water supply interrupted in most months. There were no differences in the frequency of experiencing water insecurity by sex or age; however, there were differences in the number of months participants in Walgett town experienced main water source interruption compared to participants from other areas (Tables S7 and S8).

#### 3.2.4 Other Water Security Issues Raised

When sharing further details of water security issues, participants raised all three indicators of water palatability, including appearance (*n* = 25; ‘it's brown and dirty’ (Male, 31 years)), odour (*n* = 27′ ‘horrid smell’ (Female, 61 years)) and taste (*n* = 18; ‘tastes disgusting’ (Female, 53 years)) or generally poor quality (*n* = 43). Perceived poor water quality resulted in many participants relying on bottled water for drinking and food preparation (*n* = 41). Many people reported spending $30–$50 a week on bottled water. Inability to use the town water for bathing (*n* = 15; ‘We might as well not have a wash because then you smell like the water does’ (Female, 59 years)), gardening (*n* = 12; ‘plants and lawn have died’ (Female, 61 years)), cooking (*n* = 6; ‘can't cook vegetables’ (Male, 68 years)), laundry (*n* = 4; ‘Can't wash clothes as water smells’ (Male, 72 years)) were other common complaints. One participant said they ‘buy takeaway as [they] can't prepare food with water’ (Male, 72 years). Other issues raised included hydro‐panels not working (*n* = 16), skin rashes and other acute health issues (*n* = 11).

### 3.3 Contextualising Experiences of Food and Water Insecurity in Walgett

#### 3.3.1 Sources of Food and Water

Over the previous 12 months, almost all participants (95%) reported purchasing food from the local supermarket (Table 3). Around half reported also using other local shops (52%) and the local river (44%). Participants also utilised native vegetation/bush tucker (22%), the WAMS community garden (30%), and some used other sources, such as schools (6%).

**TABLE 3:** Responses to community‐developed questions about drivers of food and water insecurity in the Aboriginal community in Walgett during the last 12 months (*n* = 251).

Questions	Frequencies (%)
Sources of food and water	(*n* = 251)
Where did you get your food from?	
Supermarket	240 (96)
Other local shops	130 (52)
Native vegetation/bush tucker	54 (22)
Local river	111 (44)
WAMS community garden	73 (29)
Other community source (PCYC, school)	15 (6)
Other	33 (13)
What was your or your household's main water source?	
Tank water	77 (31)
Bore water	72 (29)
Town water (supplied by council)	199 (79)
Bottled water	186 (74)
Source water or hydro‐panel water	95 (38)
Other	7 (3)
For participants who experienced some form of food insecurity	(*n* = 224)
What were the reasons you could not eat your desired amount or types of foods?	
Affordability of food (not enough money or food too expensive)	160 (71)
Availability of food (no food due to depleted stocks/disrupted supply chains)	140 (63)
Accessibility of food (could not get to shops/no local shops)	106 (47)
Utilisation of food (food not safe/able to be stored safely)	53 (24)
Did not have the required utilities to prepare food	
Power (electricity, gas)	57 (25)
Water	73 (33)
Other	4 (2)
Did not have the required equipment to prepare food	
Fridge	22 (10)
Freezer	14 (6)
Microwave	18 (8)
Oven	17 (8)
Pots and pans for cooking	13 (6)
Sink	11 (5)
Bench or table that can be used for food preparation	12 (5)

Participants reported using a variety of water sources in Walgett that changed throughout the year for different reasons (Table 3). In the last year, most participants (79%) mainly relied on town water (groundwater processed through the town drinking water plant). Three‐quarters (74%) also utilised bottled water in at least 1 month. Other sources of water utilised by 30%–40% of the community included tank water, untreated bore water (separate from town water system) or point‐of‐use hydro‐panels designed to capture water from the atmosphere.

#### 3.3.2 Drivers of Food and Water Insecurity

Around two‐thirds of participants attributed food insecurity to affordability (71%) and availability (63%) (Table 3). Almost half were unable to access food (47%), and one‐quarter were unable to utilise food due to safety (24%). Not having the required utilities including power (25%) and water (33%) or equipment including fridge, freezer or oven (all < 10%) to prepare food were also experienced. Almost all participants (91%) were concerned about the quality of the water. There were few meaningful differences by sex, age and location (See Tables S5–S8).

#### 3.3.3 Coping Strategies for Managing Food and Water Insecurity

**FIGURE 3:**
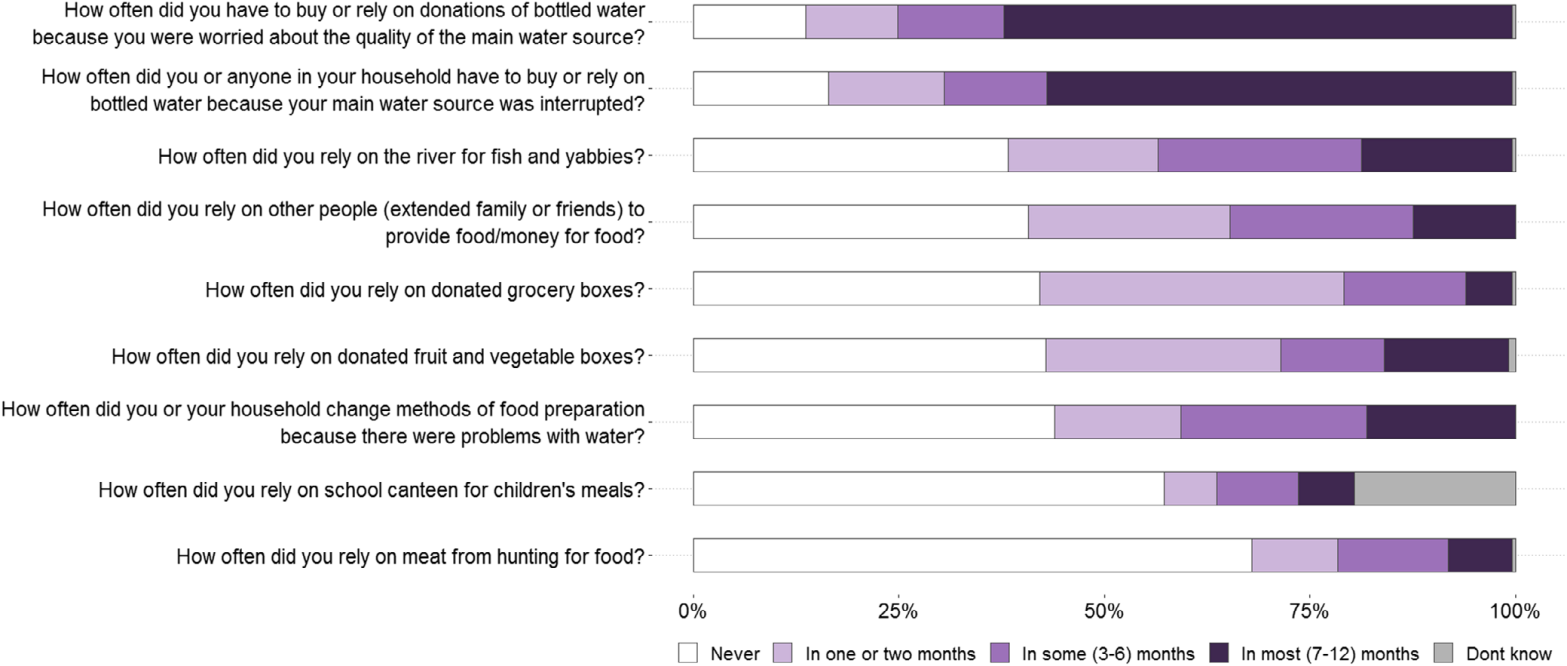
Coping strategies for food and water insecurity (*n* = 251).

The majority of participants relied on purchased or donated bottled water due to the water quality (86%) or main water source interruption (83%) in at least 1 month in the last 12 months. Other key coping strategies illustrated in Figure 3 were catching fresh fish or yabbies from the river (62%), relying on extended family and friends (59%) or donated boxes of groceries (58%) and fruit and vegetables (57%), and changing food preparation methods (56%). There were few meaningful differences by sex, age and location (See Tables S5–S8).

## 4 Discussion

This is the first study to measure food and water security levels and experiences in an Aboriginal community in Australia using culturally adapted validated survey tools. The findings confirm existing community concerns about high food and water insecurity levels in Walgett, NSW, which have a significant impact on the population, with almost half experiencing food or water insecurity.

### 4.1 Food and Water Insecurity Are More Prevalent and More Severe Than Many Communities Around the World

The prevalence of food insecurity experienced in the Aboriginal community in Walgett is unacceptable for a developed country such as Australia. Almost half of the population surveyed reported experiencing food insecurity. This is similar to, or greater than, some First Nations communities in Canada [21], and many low‐ and middle‐income countries [22]. The level of food insecurity reported in Walgett was also much higher than those reported in previous national surveys [23], as well as many subnational surveys, in Aboriginal and Torres Strait Islander communities [24]. These surveys were based on single‐question tools [25, 26], in contrast to the extensive validated HFIAS used in our study as discussed further in Section 4.3.

More than 40% of the Walgett community surveyed reported experiencing water insecurity based on the HWISE scale, which is similar to First Nations and Indigenous communities in other countries; for example, one study showed that 58% of First Nations households in Canada experienced water insecurity [27]. The level of water insecurity in Walgett is even more severe than in some communities in low‐ and middle‐income countries [28]. These findings contradict Federal government reporting that all Australians have access to safe drinking water [29]. At the time of this survey, individual‐level water insecurity had not been measured in Australia nor was there a government database to track water quality [30].

Further, publicly available water monitoring data reveal that at least 25 000 Australians do not have access to safe drinking water but many more, at least half a million Australians across 400 towns (40% of which are remote Aboriginal and Torres Strait Islander communities), do not have access to good quality drinking water [30]. Walgett town water is not meeting Australian standards for palatable, good‐quality drinking water [31] and our community survey findings reinforced that it is considered poor quality and unsafe for consumption, resulting in community distrust towards town water quality and its governance. Access to safe food and water are a fundamental human right, and the prevalence and severity of food and water insecurity in regional and remote towns in a high‐income country such as Australia is alarming.

### 4.2 Food and Water Insecurity Are Interconnected

Most existing policies in Australia and globally approach food and water issues separately [32]. Using the Aboriginal paradigm of interconnected food and water systems enabled us to demonstrate the association between food and water insecurity and better understand the interconnections. Around one‐third of the participants experienced both food and water insecurity. People experiencing water insecurity were more likely to be experiencing food insecurity, which is in line with a previous study in 25 low‐ and middle‐income communities [17].

The interconnectedness of food and water insecurity was further highlighted through issues raised by the participants and the impact on food availability, the ability to afford a healthy diet and a range of other socio‐psychological issues. For the Aboriginal community in Walgett, the quality of the river has a direct link to the health of the fish and animals and the well‐being of Aboriginal people who have traditionally eaten these foods as part of a cultural and spiritual connection to the rivers [33]. The poor quality of the river made fish and yabbies (crayfish) inedible (and when the river dried up, there was no supply), removing a key food source for the community and meaning they needed to spend additional money to replace this traditional protein source. Additionally, the town water was switched from river to bore water, which was perceived as unsafe for drinking and preparing/cooking food at home and resulted in people purchasing bottled water [34]. Some spent $30–$50 a week on bottled water which hampered their ability to be able to afford a healthy diet. Importantly, affordability was reported to be the main driver of food insecurity for most participants, which affirms previous studies on diet affordability in remote and regional populations, including Aboriginal communities [9].

### 4.3 Implications for Community and Policy

This research has identified the prevalence and severity of food and water insecurity in the Walgett community, as well as the drivers and coping strategies. Our findings reinforce other evidence that previous attempts to improve food security in Australia have had limited impact in some communities [35]. The results highlights the need to reframe the narrative in this area [36], including ensuring community ownership in establishing sustainable solutions [37].

The key to this is moving away from traditional western concepts of food and water security and centring the importance of connectedness between Aboriginal People, land, and waters towards Indigenous concepts of food and water sovereignty. Food sovereignty has previously been defined as ‘The right of people to healthy and culturally appropriate food produced through ecologically sound and sustainable methods, and their right to define their own food and agriculture systems’ [38]. Water sovereignty refers to an approach to water systems ‘based on underlying values, whereby land and water and humans and nature are integrated, and Indigenous knowledge systems are prioritised’ [12]. In this way, the narrative of food and water insecurity experiences in Australia can be reframed to encompass Indigenous knowledges and community leadership in improving food and water systems [36].

Food and water security are inextricably linked and a holistic approach to tackling these issues building on indigenous knowledge systems and community leadership is required. Further community‐led research to explore the relationship between food and water security including water quality and diet would be useful. The Australian government is currently consulting on a National Strategy for Food Security in Remote First Nations Communities. Potential options that should be considered include fiscal measures: subsidies and taxes to lower the relative price of fresh, healthy foods [39] and income supplements [40], which have been demonstrated to be effective in increasing fruit and vegetable consumption in Aboriginal and Torres Strait Islander communities [40]. The strategy should also be extended to centre Aboriginal community leadership in river and groundwater management. It should also include legislative and policy actions and resources to ensure the health, diversity and resilience of environments, support stable wild food sources and reduce threats to food sources by climate change. For this approach to be successful, and to contribute towards ‘Closing the Gap’, the initiatives need to be led by and resources need to be directed to Aboriginal Community‐Controlled Health Organisations.

Measurements of national and community level food and water insecurity in Australia have been inadequate (e.g., using single‐item surveys, not reaching all Australians) [31]. The tools used to assess food and water insecurity in this study have been used and validated extensively around the world [17]. The tools were selected as they are relatively short, measure access as well as affordability, have been used in other First Nations and Indigenous communities globally, and have a scoring system that enables comparisons between communities. We suggest the government considers these tools to measure food and water security in other parts of Australia, building on traditional knowledges and fostering community leadership to ensure the tools can be adapted to be appropriate to different settings.

### 4.4 Strengths and Limitations

A key strength of this survey was that it was codesigned with and administered by community representatives. We made culturally appropriate adaptations to globally validated survey tools [17], and pilot‐tested them in the community. A potential limitation was that we used convenience sampling; however, this was considered to be the most feasible and culturally appropriate approach. The high response rate, with around one‐quarter of the Aboriginal community participating in the survey, likely means our findings are reflective of the experiences of the broader Aboriginal community. Lastly, some of the questions may have resulted in people feeling sad or ashamed so that they moderated their responses. However, the predominantly local Aboriginal survey team shared that they felt people were answering honestly and that the findings are likely to represent their experiences.

## 5 Conclusions

Food and water insecurity in Walgett is unacceptably high, on par with the prevalence rates in low‐ and middle‐income settings and far higher than national‐level estimates in Australia. The measurement of food and water insecurity has empirically confirmed long‐standing community concerns and highlights the connection between food, water, community and Country. A holistic integrated government response and support for community‐led local initiatives are needed to improve the health and wellbeing of Aboriginal communities in Australia.

## Author Contributions

**Loretta Weatherall:** investigation, writing – original draft, writing – review and editing. **Alinta Trindall:** investigation, writing – original draft, writing – review and editing. **Trish Tonkin:** investigation, project administration, writing – review and editing. **Joseph Alvin Santos:** formal analysis, visualization, writing – review and editing. **Dori Patay:** formal analysis, writing – original draft, writing – review and editing. **Ruth McCausland:** conceptualization, methodology, funding acquisition, writing – review and editing. **Wendy Spencer:** writing – review and editing, conceptualization, methodology, project administration, funding acquisition. **Greg Leslie:** conceptualization, methodology, funding acquisition, writing – review and editing. **Eileen Baldry:** writing – review and editing, funding acquisition. **Keziah Bennett‐Brook:** writing – review and editing, funding acquisition, conceptualization, methodology. **Julieann Coombes:** writing – review and editing, funding acquisition. **Tamara Mackean:** conceptualization, methodology, funding acquisition, writing – review and editing. **Janani Shanthosh:** conceptualization, methodology, funding acquisition, writing – review and editing. **Ty Madden:** investigation, writing – review and editing. **Bruce Moore:** investigation, writing – review and editing. **Ann‐Marie Deane:** investigation, writing – review and editing. **Niall Earle:** investigation, writing – review and editing. **Christine Corby GDip:** conceptualization, methodology, writing – review and editing, funding acquisition. **Melissa Nathan:** project administration, writing – review and editing. **Sera L. Young:** conceptualization, methodology, writing – review and editing. **Emalie Rosewarne:** conceptualization, methodology, formal analysis, writing – original draft, writing – review and editing, supervision, project administration. **Jacqui Webster:** conceptualization, methodology, formal analysis, investigation, writing – original draft, writing – review and editing, supervision, project administration, funding acquisition.

## Disclosure

This research was funded by a National Health and Medical Research Council of Australia IDEAS grant (#2003862). JW is supported in her work through a National Health and Medical Research Council Investigator Grant (#2018015). This work has not been published elsewhere in part or in full.

## Ethics Statement

The study was conducted in accordance with the Declaration of Helsinki. Ethical approval was obtained from the Aboriginal Health and Medical Research Council of New South Wales (1781/20) and was ratified by the University of New South Wales Human Research Ethics Committee. All participating adults provided written consent.

## Conflicts of Interest

The authors declare no conflicts of interest.

## Supporting information


Table S1.

Table S2.

Table S3.

Table S4.

Table S5.
Table S6.
Table S7.
Table S8.

## Data Availability

The data that support the findings of this study are available on request from the corresponding author. The data are not publicly available due to privacy or ethical restrictions.

## Endnote

^1^
Whilst 2021 census data is available it is currently contested and likely under‐represents the Aboriginal population so we have used the 2016 census data.
