# Supplementary material for: Measuring Food and Water Security in an Aboriginal Community in Regional Australia
Source: Aust J Rural Health. 2025 Jan 12;33(1):e13214. doi: 10.1111/ajr.13214 (PMC11725624; doi:10.1111/ajr.13214)
Supplement: Supplementary file 2 — Table S2. [file AJR-33-0-s005.docx]

**Supplementary S2: Food and Water Security Questionnaire**

Introduction

The following questions ask about food security and water security. Just a reminder that all information will be treated in confidence and anonymised in any reports.

Demographics

**Read:** First, we will ask you a few general questions about yourself.

## 1.1 What gender do you identify as?

Male

Female

Non-binary

Other

## 1.2 How old are you?

____________________

## 1.3 Are you of Aboriginal origin?

No

Yes

## 1.4 Which of the following best describes your highest level of education?

Secondary education (Years 7-12) or below

Tertiary education (University)

Postgraduate or higher

## 1.5 How many people do you usually live with?

Live alone

Live with partner

Shared household (2-5 people)

Shared household (6-8 people)

Shared household (more than 8 people)

## 1.6 How many people do you usually eat with?

Eat alone

Eat with partner

Shared household (2-5 people)

Shared household (6-8 people)

Shared household (more than 8 people)

## 1.7 Which of the following best describes your role?

Responsible for both shopping and cooking most of the time

Responsible for shopping for food most of the time

Responsible for cooking most of the time

Not responsible for shopping and cooking/Eat with family most of the time

Food Security

**Read:** Next I will ask you about your experience with food. For each experience, we want to know in how many months it happened to you during the last 12 months. Even if it only happened once during a month, we’d like you to count that month (read items).

# Australian Bureau of Statistics

## 2.1 In the last 12 months, have you or anyone in your household run out of food?

Yes

No

## 2.1.1 What was the reason for this?

Did not have enough money to purchase more

Food not available to buy

Other, please specify

# Household Food Insecurity Access Scale (HFIAS)

**Anxiety and uncertainty about the household food supply**

## 2.2 In the last 12 months, did you worry that your household would not have enough food?

Never

In one or two months

In some (2-6) months

In most (7-12) months

Don’t know

**Insufficient Quality (includes variety and preferences of the type of food)**

## 2.3 In the last 12 months, were you or any household member not able to eat the kinds of foods you preferred?

Never

In one or two months

In some (2-6) months

In most (7-12) months

Don’t know

## 2.4 In the last 12 months, did you or any household member have to eat a limited variety of foods?

Never

In one or two months

In some (2-6) months

In most (7-12) months

Don’t know

## 2.5 In the last 12 months, did you or any household member have to eat some foods that you really did not want to eat because you could not obtain other types of food?

Never

In one or two months

In some (2-6) months

In most (7-12) months

Don’t know

**Insufficient food intake and its physical consequences**

## 2.6 In the last 12 months, did you or any household member have to eat a smaller meal than you felt you needed because there was not enough food?

Never

In one or two months

In some (2-6) months

In most (7-12) months

Don’t know

## 2.7 In the last 12 months, did you or any household member have to eat fewer meals in a day because there was not enough food?

Never

In one or two months

In some (2-6) months

In most (7-12) months

Don’t know

**Lack of food**

## 2.8 In the last 12 months, was there ever no food to eat of any kind in your household?

Never

In one or two months

In some (2-6) months

In most (7-12) months

Don’t know

## 2.9 In the last 12 months, did you or any household member go to sleep at night hungry because there was not enough food?

Never

In one or two months

In some (2-6) months

In most (7-12) months

Don’t know

## 2.10 In the last 12 months, did you or any household member go a whole day and night without eating anything because there was not enough food?

Never

In one or two months

In some (2-6) months

In most (7-12) months

Don’t know

# Supplementary Questions

## 2.11 In the last 12 months, what were the reasons you could not eat your desired amount or types of foods? (tick all that apply)

Affordability of food

Not enough money for food

Foods too expensive

Availability of food

Lack of food available in local shop to buy (stocks depleted, disrupted supply chains)

Accessibility of food

Could not get to local shop to buy food

Could not get to any shop to buy food when there was no local shop

Utilisation of food

Food was not safe to consume (e.g. expired)

Food was not able to be safely stored or prepared (e.g. could not be washed)

Did not have the required utilities to prepare food

Power e.g. electricity, gas

Water

Other, please specify

Did not have the required equipment to prepare food

Fridge

Freezer

Microwave

Oven

Pots and pans for cooking

Sink

Bench or table that can be used for food preparation

Other, please specify

**2.12 In the last 12 months, where did you get your food from? (tick all that apply)**

Supermarket

Native vegetation/bush tucker

Local river

WAMS community garden

Other community source e.g. PCYC, school

Other, please specify

## 2.13 Still thinking about the last 12 months, how often did you rely on the following:

1. **other people (e.g. extended family or friends) to provide food/money for food**

Never

In one or two months

In some (2-6) months

In most (7-12) months

Don’t know

1. **school canteen for children’s meals**

Never

In one or two months

In some (2-6) months

In most (7-12) months

Don’t know

1. **the river for fish and seafood**

Never

In one or two months

In some (2-6) months

In most (7-12) months

Don’t know

1. **meat from hunting or roadkill**

Never

In one or two months

In some (2-6) months

In most (7-12) months

Don’t know

1. **donated grocery boxes**

Never

In one or two months

In some (2-6) months

In most (7-12) months

Don’t know

1. **donated fruit and vegetable boxes**

Never

In one or two months

In some (2-6) months

In most (7-12) months

Don’t know

**Food Security**

Water Security

(Read:) Next I will ask you about your experience with water. For each experience, we want to know in how many months it happened to you during the last 12 months. Even if it only happened once during a month, we’d like you to count that month (read items).

Interviewer: Repeat scale as necessary after the first time. If respondent says “in every month” code as “in most months (7-12)”

# Household Water Insecurity Experiences Scale († Additional question)

**Anxiety and uncertainty about household water supply**

## 3.1 In the last 12 months, how often did you or anyone in your household worry that you would not have enough water for all of your needs?

Never

In one or two months

In some (2-6) months

In most (7-12) months

Don’t know

**Insufficient drinking water quantity**

## 3.2† In the last 12 months, what was your or your household’s main water source?

Tank water

Bore water

Town water (supplied by council)

Bottled water

Source water or hydro-panel water

Other, please specify ___________

## 3.3 In the last 12 months, how often was your or your household’s main water source interrupted or limited in any way (e.g. low or no water pressure, less water than expected, source dried up)?

Never

In one or two months

In some (2-6) months

In most (7-12) months

Don’t know

## 3.4† In the last 12 months, how often did you or anyone in your household have to buy or rely on bottled water (donations or bought) because your main water source (tap/bore) was interrupted?

Never

In one or two months

In some (2-6) months

In most (7-12) months

Don’t know

## 3.5 In the last 12 months, how often did you or anyone in your household NOT have enough water to drink as you would have liked?

Never

In one or two months

In some (2-6) months

In most (7-12) months

Don’t know

## 3.6 In the last 12 months, how often did you or anyone in your household change what you ate because there were problems with water (e.g., for washing foods, cooking, etc.)?

Never

In one or two months

In some (2-6) months

In most (7-12) months

Don’t know

## 3.7† In the last 12 months, how often did you or your household change methods of food preparation because there were problems with water (e.g., for washing foods, cooking, etc.)?

Never

In one or two months

In some (2-6) months

In most (7-12) months

Don’t know

**Insufficient water for hygiene and household purposes**

## **3.8 In the last 12 months, how often have you or anyone in your household had to go without washing hands after dirty activities (e.g.,** defecating or changing diapers, cleaning animal dung) because of problems with water?

Never

In one or two months

In some (2-6) months

In most (7-12) months

Don’t know

## 3.9 In the last 12 months, how often have you or anyone in your household had to go without washing their body because of problems with water (e.g., not enough water, dirty, unsafe)?

Never

In one or two months

In some (2-6) months

In most (7-12) months

Don’t know

## 3.10 In the last 12 months, how often has problems with water meant that clothes could not be washed?

Never

In one or two months

In some (2-6) months

In most (7-12) months

Don’t know

## 3.11 In the last 12 months, how often have you or anyone in your household had to change schedules or plans due to problems with your water situation? (Activities that may have been interrupted include caring for others, doing household chores, agricultural work, income-generating activities, sleeping, etc.)

Never

In one or two months

In some (2-6) months

In most (7-12) months

Don’t know

**Lack of water**

## 3.12 In the last 12 months, how often did you or anyone in your household go to sleep thirsty because there was no drinkable water to drink?

Never

In one or two months

In some (2-6) months

In most (7-12) months

Don’t know

## 3.12 In the last 12 months, how often did you or anyone in your household have no usable or drinkable water whatsoever?

Never

In one or two months

In some (3-6) months

In most (7-12) months

Don’t know

**Feelings about water security**

## 3.14 In the last 12 months, how often did you or anyone in your household feel angry about your water situation?

Never

In one or two months

In some (2-6) months

In most (7-12) months

Don’t know

## 3.15 In the last 12 months, how often have problems with water caused you or anyone in your household to feel ashamed/excluded/stigmatized?

Never

In one or two months

In some (2-6) months

In most (7-12) months

Don’t know

# Supplementary questions

**Insufficient water quality**

## **3.16 In the last 12 months, how often were you concerned about the quality (taste, smell, contamination) of your** main water source?

Never

In one or two months

In some (2-6) months

In most (7-12) months

Don’t know

## 3.17 In **the last 12 months, how often did you have to buy or rely on donations of bottled water because you were worried about the quality (taste/smell/contamination) of the main water source** (tap/bore)?

Never

In one or two months

In some (2-6) months

In most (7-12) months

Don’t know

## 3.18 In **the last 12 months, how often did you NOT have enough water for indoor use such as cooking,** bathing, washing and cleaning as you would have liked?

Never

In one or two months

In some (2-6) months

In most (7-12) months

Don’t know

## 3.19 In **the last 12 months, how often did you NOT have enough water for outdoor use (gardening) as** would have liked?

Never

In one or two months

In some (2-6) months

In most (7-12) months

Don’t know
