# Supplementary material for: Measuring Food and Water Security in an Aboriginal Community in Regional Australia
Source: Aust J Rural Health. 2025 Jan 12;33(1):e13214. doi: 10.1111/ajr.13214 (PMC11725624; doi:10.1111/ajr.13214)
Supplement: Supplementary file 3 — Table S3. [file AJR-33-0-s003.docx]

**Supplementary Table S3.** Mapping of questions from the community surveys to validated, global tools for the calculation of HFIA score

| Q | **From Coates J et al 2007 study** | **Walgett Food and Water Security survey** |
| --- | --- | --- |
| **1** | In the past four weeks, did you worry that your household would not have enough food? | In the last 12 months, did you worry that your household would not have enough food? |
| **2** | In the past four weeks, were you or any household member not able to eat the kinds of foods you preferred because of a lack of resources? | In the last 12 months, were you or any household member not able to eat the kinds of foods you preferred? |
| **3** | In the past four weeks, did you or any household member have to eat a limited variety of foods due to a lack of resources? | In the last 12 months, did you or any household member have to eat a limited variety of foods? |
| **4** | In the past four weeks, did you or any household member have to eat some foods that you really did not want to eat because of a lack of resources to obtain other types of food? | In the last 12 months, did you or any household member have to eat some foods that you really did not want to eat because you could not obtain other types of food? |
| **5** | In the past four weeks, did you or any household member have to eat a smaller meal than you felt you needed because there was not enough food? | In the last 12 months, did you or any household member have to eat a smaller meal than you felt you needed because there was not enough food? |
| **6** | In the past four weeks, did you or any household member have to eat fewer meals in a day because there was not enough food? | In the last 12 months, did you or any household member have to eat fewer meals in a day because there was not enough food? |
| **7** | In the past four weeks, was there ever no food to eat of any kind in your household because of lack of resources to get food? | In the last 12 months, was there ever no food to eat of any kind in your household? |
| **8** | In the past four weeks, did you or any household member go to sleep at night hungry because there was not enough food? | In the last 12 months, did you or any household member go to sleep at night hungry because there was not enough food? |
| **9** | In the past four weeks, did you or any household member go a whole day and night without eating anything because there was not enough food? | In the last 12 months, did you or any household member go a whole day and night without eating anything because there was not enough food? |
