# Supplementary material for: Measuring Food and Water Security in an Aboriginal Community in Regional Australia
Source: Aust J Rural Health. 2025 Jan 12;33(1):e13214. doi: 10.1111/ajr.13214 (PMC11725624; doi:10.1111/ajr.13214)
Supplement: Supplementary file 4 — Table S4. [file AJR-33-0-s001.docx]

**Supplementary Table S4.** Mapping of questions from the community surveys to validated, global tools for the calculation of HWISE score

| Q | **From Young S et al 2019 study** | **Walgett Food and Water Security survey** |
| --- | --- | --- |
| **1** | In the last 4 weeks, how frequently did you or anyone in your household worry you would not have enough water for all of your household needs? | In the last 12 months, how often did you or anyone in your household worry that you would not have enough water for all of your needs? |
| **2** | In the last 4 weeks, how frequently has your main water source been interrupted or limited (eg, water pressure, less water than expected, river dried up)? | In the last 12 months, how often was your or your household's main water source interrupted or limited in any way (e.g. low or no water pressure, less water than expected, source dried up)? |
| **3** | In the last 4 weeks, how frequently have problems with water meant that clothes could not be washed? | In the last 12 months, how often has problems with water meant that clothes could not be washed? |
| **4** | In the last 4 weeks, how frequently have you or anyone in your household had to change schedules or plans due to problems with your water situation? (Activities that may have been interrupted include caring for others, doing household chores, agricultural work, income-generating activities, etc.) | In the last 12 months, how often have you or anyone in your household had to change schedules or plans due to problems with your water situation? (Activities that may have been interrupted include caring for others, doing household chores, income-generating activities) |
| **5** | In the last 4 weeks, how frequently have you or anyone in your household had to change what was being eaten because there were problems with water (eg, for washing foods, cooking, etc.)? | In the last 12 months, how often did you or anyone in your household change what you ate because there were problems with water (e.g. for washing foods, cooking)? |
| **6** | In the last 4 weeks, how frequently have you or anyone in your household had to go without washing hands after dirty activities (eg, defecating or changing diapers, cleaning animal dung) because of problems with water? | In the last 12 months, how often have you or anyone in your household had to go without washing hands after dirty activities (e.g. going to the toilet or changing diapers, cleaning up after animals) because of problems with water? |
| **7** | In the last 4 weeks, how frequently have you or anyone in your household had to go without washing their body because of problems with water (eg, not enough water, dirty, unsafe)? | In the last 12 months, how often have you or anyone in your household had to go without washing their body because of problems with water (e.g. not enough water, dirty, unsafe)? |
| **8** | In the last 4 weeks, how frequently has there not been as much water to drink as you would like for you or anyone in your household? | In the last 12 months, how often did you or anyone in your household not have enough water to drink as you would have liked? |
| **9** | In the last 4 weeks, how frequently did you or anyone in your household feel angry about your water situation? | In the last 12 months, how often did you or anyone in your household feel angry about your water situation? |
| **10** | In the last 4 weeks, how frequently have you or anyone in your household gone to sleep thirsty because there wasn’t any water to drink? | In the last 12 months, how often did you or anyone in your household go to sleep thirsty because there was no drinkable water to drink? |
| **11** | In the last 4 weeks, how frequently has there been no useable or drinkable water whatsoever in your household? | In the last 12 months, how often did you or anyone in your household have no usable or drinkable water whatsoever? |
| **12** | In the last 4 weeks, how frequently have problems with water caused you or anyone in your household to feel ashamed/excluded/stigmatised? | In the last 12 months, how often have problems with water caused you or anyone in your household to feel ashamed or excluded? |
