# Supplementary material for: Measuring Food and Water Security in an Aboriginal Community in Regional Australia
Source: Aust J Rural Health. 2025 Jan 12;33(1):e13214. doi: 10.1111/ajr.13214 (PMC11725624; doi:10.1111/ajr.13214)
Supplement: Supplementary file 5 — Table S5. Table S6. Table S7. Table S8. [file AJR-33-0-s004.docx]

Supplementary Table S5. Food insecurity indicators experienced by participants during the last 12 months, overall, by sex, age and location (n, %)

| **QUESTIONS** | **OVERALL**  (*n*=251) | **BY SEX** | | **BY AGE GROUP** | | **BY LOCATION ^a^** | |
| --- | --- | --- | --- | --- | --- | --- | --- |
|  |  | **Males**  (*n*=112) | **Females**  (*n*=139) | **18 to 44 years**  (*n*=116) | **45 years and up**  (*n*=135) | **Walgett town**  (*n*=203) | **Other areas ^b^**  (*n*=46) |
| **Have you or anyone in your household run out of food?** | | | | | | | |
| Never  In one or two months  In some (3-6) months  In most (7-12) months  Don’t know | 126 (50.6)  56 (22.5)  45 (18.1)  19 (7.6)  3 (1.2) | 57 (51.8)  19 (17.3)  25 (22.7)  8 (7.3)  1 (0.9) | 69 (49.6)  37 (26.6)  20 (14.4)  11 (7.9)  2 (1.4) | 64 (56.1)  21 (18.4)  21 (18.4)  8 (7.0)  0 (0.0) | 62 (45.9)  35 (25.9)  24 (17.8)  11 (8.2)  3 (2.2) | 96 (47.5)  47 (23.3)  40 (19.8)  17 (8.4)  2 (1) | 28 (62.2)  9 (20.0)  5 (11.1)  2 (4.4)  1 (2.2) |
| **Did you worry that your household would not have enough food?** | | | | | | | |
| Never  In one or two months  In some (3-6) months  In most (7-12) months  Don’t know | 83 (33.1)  53 (21.1)  57 (22.7)  54 (21.5)  4 (1.6) | 43 (38.4)  20 (17.9)  24 (21.4)  21 (18.8)  4 (3.6) | 40 (28.8)  33 (23.7)  33 (23.7)  33 (23.7)  0 (0.0) | 39 (33.6)  25 (21.66)  30 (25.9)  19 (16.4)  3 (2.6) | 44 (32.6)  28 (20.7)  27 (20.0)  35 (25.9)  1 (0.7) | 66 (32.5)  40 (19.7)  45 (22.2)  49 (24.1)  3 (1.5) | 15 (32.6)  13 (28.3)  12 (26.1)  5 (10.9)  1 (2.1) |
| **Were you or any household member not able to eat the kinds of foods you preferred?** | | | | | | | |
| Never  In one or two months  In some (3-6) months  In most (7-12) months  Don’t know | 87 (34.8)  51 (20.4)  69 (27.6)  41 (16.4)  2 (0.8) | 41 (36.6)  23 (20.5)  30 (26.8)  16 (14.3)  2 (1.8) | 46 (33.3)  28 (20.1)  39 (28.3)  25 (18.1)  0 (0.0) | 41 (35.7)  30 (26.1)  31 (27)  12 (10.4)  1 (0.9) | 46 (34.1)  21 (15.6)  38 (28.2)  29 (21.5)  1 (0.7) | 75 (37.1)  41 (20.3)  51 (25.3)  34 (16.8)  1 (0.5) | 11 (23.9)  10 (21.7)  18 (39.1)  7 (15.2)  0 (0.0) |
| **Did you or any household member have to eat a limited variety of foods?** | | | | | | | |
| Never  In one or two months  In some (3-6) months  In most (7-12) months  Don’t know | 96 (38.3)  44 (17.5)  61 (24.3)  50 (19.9)  0 (0.0) | 41 (36.6)  21 (18.8)  32 (28.6)  18 (16.1)  0 (0.0) | 55 (39.6)  23 (16.6)  29 (20.9)  32 (23.0)  0 (0.0) | 50 (43.1)  20 (17.2)  28 (24.1)  18 (15.5)  0 (0.0) | 46 (34.1)  24 (17.8)  33 (24.4)  32 (23.7)  0 (0.0) | 81 (39.9)  34 (16.8)  43 (21.2)  45 (22.2)  0 (0.0) | 14 (30.4)  10 (21.7)  17 (36.9)  5 (10.9)  0 (0.0) |
| **Did you or any household member have to eat some foods that you really did not want to eat because you could not obtain other types of food?** | | | | | | | |
| Never  In one or two months  In some (3-6) months  In most (7-12) months  Don’t know | 103 (41.0)  49 (19.5)  58 (23.1)  40 (15.9)  1 (0.4) | 48 (42.9)  22 (19.6)  25 (22.3)  17 (15.2)  0 (0.0) | 55 (39.6)  27 (19.4)  33 (23.7)  23 (16.6)  1 (0.7) | 53 (45.7)  22 (19)  29 (25)  12 (10.3)  0 (0.0) | 50 (37.0)  27 (20)  29 (21.5)  28 (20.7)  1 (0.7) | 87 (42.9)  40 (19.7)  43 (21.2)  33 (16.3)  0 (0.0) | 15 (32.6)  9 (19.6)  14 (30.4)  7 (15.2)  1 (2.2) |
| **Did you or any household member have to eat a smaller meal than you felt you needed because there was not enough food? ^3^** | | | | | | | |
| Never  In one or two months  In some (3-6) months  In most (7-12) months  Don’t know | 105 (41.8)  44 (17.5)  54 (21.5)  48 (19.1)  0 (0.0) | 46 (41.1)  22 (19.6)  26 (23.2)  18 (16.1)  0 (0.0) | 59 (42.5)  22 (15.8)  28 (20.1)  30 (21.6)  0 (0.0) | 56 (48.3)  18 (15.5)  24 (20.7)  18 (15.5)  0 (0.0) | 49 (36.3)  26 (19.3)  30 (22.2)  30 (22.2)  0 (0.0) | 92 (45.3)  36 (17.7)  36 (17.7)  39 (19.2)  0 (0.0) | 12 (26.1)  8 (17.4)  18 (39.1)  8 (17.4)  0 (0.0) |
| **Did you or any household member have to eat fewer meals in a day because there was not enough food? ^3^** | | | | | | | |
| Never  In one or two months  In some (3-6) months  In most (7-12) months  Don’t know | 123 (49.0)  47 (18.7)  54 (21.5)  26 (10.4)  1 (0.4) | 48 (42.9)  28 (25.0)  23 (20.5)  13 (11.6)  0 (0.0) | 75 (54)  19 (13.7)  31 (22.3)  13 (9.4)  1 (0.7) | 65 (53)  23 (20)  19 (16.4)  9 (7.8)  0 (0.0) | 58 (43)  24 (17.8)  35 (25.9)  17 (12.6)  1 (0.7) | 107 (52.7)  35 (17.2)  38 (18.7)  22 (10.8)  1 (0.5) | 15 (32.6)  12 (26.1)  16 (34.8)  3 (6.5)  0 (0.0) |
| **Was there ever no food to eat of any kind in your household? ^1^** | | | | | | | |
| Never  In one or two months  In some (3-6) months  In most (7-12) months  Don’t know | 175 (69.7)  36 (14.3)  31 (12.4)  8 (3.2)  1 (0.4) | 73 (65.2)  24 (21.4)  12 (10.7)  2 (1.8)  1 (0.9) | 102 (73.4)  12 (8.6)  19 (13.7)  6 (4.3)  0 (0.0) | 84 (72.4)  18 (15.5)  13 (11.2)  1 (0.9)  0 (0.0) | 91 (67.4)  18 (13.3)  18 (13.3)  7 (5.2)  1 (0.7) | 140 (69)  31 (15.3)  25 (12.3)  6 (3)  1 (0.5) | 33 (71.7)  5 (10.9)  6 (13)  2 (4.4)  0 (0.0) |
| **Did you or any household member go to sleep at night hungry because there was not enough food? ^1^** | | | | | | | |
| Never  In one or two months  In some (3-6) months  In most (7-12) months  Don’t know | 178 (71.2)  33 (13.2)  28 (11.2)  11 (4.4)  0 (0.0) | 71 (64)  23 (20.7)  13 (11.7)  4 (3.6)  0 (0.0) | 107 (77)  10 (7.2)  15 (10.8)  7 (5.0) 0 (0.0) | 86 (74.8)  12 (10.4)  15 (13)  2 (1.7)  0 (0.0) | 92 (68.2)  21 (15.6)  13 (9.6)  9 (6.7)  0 (0.0) | 142 (70.3)  28 (13.9)  23 (11.4)  9 (4.5)  0 (0.0) | 34 (73.9)  5 (10.9)  5 (10.9)  2 (4.4)  0 (0.0) |
| **Did you or any household member go a whole day and night without eating anything because there was not enough food? ^1^** | | | | | | | |
| Never  In one or two months  In some (3-6) months  In most (7-12) months  Don’t know | 195 (77.7)  31 (12.4)  19 (7.6)  6 (2.4)  0 (0.0) | 81 (72.3)  20 (17.9)  11 (9.8)  0 (0.0)  0 (0.0) | 114 (82)  11 (7.9)  8 (5.8)  6 (4.3)  0 (0.0) | 95 (81.9)  10 (8.6)  9 (7.8)  2 (1.7)  0 (0.0) | 100 (74.1)  21 (15.6)  10 (7.4)  4 (3)  0 (0.0) | 156 (76.9)  27 (13.3)  16 (7.9)  4 (2)  0 (0.0) | 37 (80.4)  4 (8.7)  3 (6.5)  2 (4.4)  0 (0.0) |

^a^ Analysis by location excludes two respondents with unknown location. ^b^ Other areas include Gingie Village, Namoi Village, and out of town Walgett area. ^1^ Significant difference by sex at p<0.05. ^2^ Significant difference by age group at p<0.05. ^3^ Significant difference by location at p<0.05.

**Supplementary Table S6.** Additional questions (developed through community consultation) to understand experiences of food insecurity during the last 12 months, overall, by sex, age and location (n, %)

| **QUESTIONS** | **OVERALL**  (*n*=251) | **BY SEX** | | **BY AGE GROUP** | | **BY LOCATION ^a^** | |
| --- | --- | --- | --- | --- | --- | --- | --- |
|  |  | **Males**  (*n*=112) | **Females**  (*n*=139) | **18 to 44 years**  (*n*=116) | **45 years and up**  (*n*=135) | **Walgett town**  (*n*=203) | **Other areas ^b^**  (*n*=46) |
| **For participants who experienced some form of food insecurity** | **(*n=*224)** | **(*n*=98)** | **(*n*=126)** | **(*n*=101)** | **(*n*=123)** | **(*n*=179)** | **(*n*=43)** |
| **What were the reasons you could not eat your desired amount or types of foods? (select as many as relevant)** | | | | | | | |
| Affordability of food (not enough money or food too expensive) ^3^ | 160 (71.4) | 73 (74.5) | 87 (69.1) | 69 (68.3) | 91 (74.0) | 122 (68.2) | 36 (83.7) |
| Availability of food (no food on shelves due to depleted stocks/disrupted supply chains) ^1^ | 140 (62.5) | 69 (70.4) | 71 (56.4) | 62 (61.4) | 78 (63.4) | 108 (60.3) | 31 (72.1) |
| Accessibility of food (could not get to shops/no local shops) ^3^ | 106 (47.3) | 53 (54.1) | 53 (42.1) | 47 (46.5) | 59 (48.0) | 71 (39.7) | 35 (81.4) |
| Utilisation of food (food not safe/able to be stored safely) ^3^ | 53 (23.7) | 22 (22.5) | 31 (24.6) | 23 (22.8) | 30 (24.4) | 29 (16.2) | 24 (55.8) |
| **Did not have the required utilities to prepare food (select as many as relevant)** | | | | | | | |
| Power (electricity, gas) | 57 (25.5) | 24 (24.5) | 33 (26.2) | 23 (22.8) | 34 (27.6) | 44 (24.6) | 11 (25.6) |
| Water ^2^ | 73 (32.6) | 32 (32.7) | 41 (32.5) | 26 (25.7) | 47 (38.2) | 57 (31.8) | 15 (34.9) |
| Other | 4 (1.8) | 3 (3.1) | 1 (0.8) | 1 (1.0) | 3 (2.4) | 3 (1.7) | 1 (2.3) |
| **Did not have the required equipment to prepare food (select as many as relevant)** | | | | | | | |
| Fridge ^1^ | 22 (9.8) | 14 (14.3) | 8 (6.4) | 9 (8.9) | 13 (10.6) | 16 (8.9) | 5 (11.6) |
| Freezer ^1^ | 14 (6.3) | 11 (11.2) | 3 (2.4) | 5 (5.0) | 9 (7.3) | 10 (5.6) | 3 (7.0) |
| Microwave | 18 (8.0) | 10 (10.2) | 8 (6.4) | 7 (6.9) | 11 (8.9) | 14 (7.8) | 3 (7.0) |
| Oven | 17 (7.6) | 8 (8.2) | 9 (7.1) | 7 (6.9) | 10 (8.1) | 13 (7.3) | 3 (7.0) |
| Pots and pans for cooking | 13 (5.8) | 6 (6.1) | 7 (5.6) | 6 (5.9) | 7 (5.7) | 10 (5.6) | 2 (4.7) |
| Sink | 11 (4.9) | 7 (7.1) | 4 (3.2) | 4 (4.0) | 7 (5.7) | 8 (4.5) | 2 (4.7) |
| Bench or table that can be used for food preparation | 12 (5.4) | 6 (6.1) | 6 (4.8) | 6 (5.9) | 6 (4.9) | 9 (5.0) | 2 (4.7) |
| Other | 8 (3.6) | 4 (4.1) | 4 (3.2) | 4 (4.0) | 4 (3.3) | 7 (3.9) | 1 (2.3) |
| **Where did you get your food from? (select as many as relevant)** | | | | | | | |
| Supermarket | 240 (95.6) | 107 (95.5) | 133 (95.7) | 112 (96.6) | 128 (94.8) | 195 (96.0) | 43 (93.5) |
| Other local shops | 130 (51.8) | 60 (53.6) | 70 (50.4) | 54 (46.6) | 76 (56.3) | 111 (54.7) | 18 (39.1) |
| Native vegetation/bush tucker ^1^ | 54 (21.5) | 33 (29.5) | 21 (15.1) | 25 (21.6) | 29 (21.5) | 44 (21.7) | 9 (19.6) |
| Local river | 111 (44.2) | 56 (50.0) | 55 (39.6) | 48 (41.4) | 63 (46.7) | 93 (45.8) | 17 (37.0) |
| WAMS community garden ^2^ | 73 (29.1) | 38 (33.9) | 35 (25.2) | 23 (19.8) | 50 (37.0) | 58 (28.6) | 14 (30.4) |
| Other community source (PCYC, school) | 15 (66.0) | 4 (3.6) | 11 (7.9) | 8 (6.9) | 7 (5.2) | 15 (7.4) | 0 (0.0) |
| Other ^2^ | 33 (13.2) | 15 (13.4) | 18 (13.0) | 9 (7.8) | 24 (17.8) | 27 (13.3) | 5 (10.9) |
| **How often did you rely on other people (extended family or friends) to provide food/money for food?** | | | | | | | |
| Never  In one or two months  In some (3-6) months  In most (7-12) months  Don’t know | 95 (40.8)  57 (24.5)  52 (22.3)  29 (12.5)  0 (0.00) | 37 (36.3)  23 (22.6)  24 (23.5)  18 (17.7)  0 (0.00) | 58 (44.3)  34 (26.0)  28 (21.4)  11 (8.4)  0 (0.00) | 45 (42.5)  24 (22.6)  28 (26.4)  9 (8.5)  0 (0.00) | 50 (39.4)  33 (26.0)  24 (18.9)  20 (15.8)  0 (0.0) | 70 (37.4)  51 (27.3)  41 (21.9)  25 (13.4)  0 (0.0) | 25 (56.8)  6 (13.6)  10 (22.7)  3 (6.8)  0 (0.0) |
| **How often did you rely on school canteen for children’s meals? ^2^** | | | | | | | |
| Never  In one or two months  In some (3-6) months  In most (7-12) months  Don’t know or NA | 126 (57.3)  14 (6.4)  22 (10.0)  15 (6.8)  43 (19.6) | 50 (51.6)  7 (7.2)  11 (11.3)  6 (6.2)  23 (23.7) | 76 (61.8)  7 (5.7)  11 (8.9)  9 (7.3)  20 (16.3) | 59 (56.2)  6 (5.7)  18 (17.1)  8 (7.6)  14 (13.3) | 67 (58.3)  8 (7.0)  4 (3.5)  7 (6.1)  29 (25.2) | 95 (53.4)  12 (6.7)  20 (11.2)  14 (7.9)  37 (20.8) | 31 (77.5)  2 (5.0)  2 (5.0)  1 (2.5)  4 (10.0) |
| **How often did you rely on the river for fish and seafood? ^1^** | | | | | | | |
| Never  In one or two months  In some (3-6) months  In most (7-12) months  Don’t know | 88 (38.3)  42 (18.3)  57 (24.8)  42 (18.3)  1 (0.4) | 30 (30.0)  18 (18.0)  26 (26.0)  26 (26.0)  0 (0.00) | 58 (44.6)  24 (18.5)  31 (23.9)  16 (12.3)  1 (0.8) | 45 (42.5)  17 (16.0)  26 (24.5)  17 (16.0)  1 (0.9) | 43 (34.7)  25 (20.2)  31 (25.0)  25 (20.2)  0 (0.0) | 75 (40.8)  35 (19.0)  40 (21.7)  33 (17.9)  1 (0.5) | 13 (29.6)  6 (13.6)  17 (38.6)  8 (18.2)  0 (0.0) |
| **How often did you rely on meat from hunting or roadkill for food? ^1^** | | | | | | | |
| Never  In one or two months  In some (3-6) months  In most (7-12) months  Don’t know | 157 (68.0)  24 (10.4)  31 (13.4)  18 (7.8)  1 (0.4) | 57 (55.9)  16 (15.7)  18 (17.7)  11 (10.8)  0 (0.00) | 100 (77.5)  8 (6.2)  13 (10.1)  7 (5.4)  1 (0.8) | 75 (70.8)  11 (10.4)  13 (12.3)  6 (5.7)  1 (0.9) | 82 (65.6)  13 (10.4)  18 (14.4)  12 (9.6)  0 (0.0) | 130 (70.3)  18 (9.8)  21 (11.4)  15 (8.1)  1 (0.5) | 26 (59.1)  6 (13.6)  9 (20.5)  3 (6.8)  0 (0.0) |
| **How often did you rely on donated grocery boxes?** | | | | | | | |
| Never  In one or two months  In some (3-6) months  In most (7-12) months  Don’t know | 97 (42.2)  85 (37.0)  34 (14.8)  13 (5.7)  1 (0.4) | 32 (31.7)  45 (44.6)  16 (15.8)  7 (6.9)  1 (1.0) | 65 (50.4)  40 (31.0)  18 (14.0)  6 (4.7)  0 (0.00) | 48 (46.2)  37 (35.6)  15 (14.4)  3 (2.9)  1 (1.0) | 49 (38.9)  48 (38.1)  19 (15.1)  10 (7.9)  0 (0.0) | 82 (44.6)  65 (35.3)  25 (13.6)  11 (6.0)  1 (0.5) | 14 (31.8)  20 (45.5)  8 (18.2)  2 (4.6)  0 (0.0) |
| **How often did you rely on donated fruit and vegetable boxes? ^3^** | | | | | | | |
| Never  In one or two months  In some (3-6) months  In most (7-12) months  Don’t know | 99 (42.7)  66 (28.6)  29 (12.6)  35 (15.2)  2 (0.9) | 36 (35.6)  34 (33.7)  13 (12.9)  16 (15.8)  2 (2.0) | 63 (48.5)  32 (24.6)  16 (12.3)  19 (14.6)  0 (0.00) | 48 (45.7)  34 (32.4)  12 (11.4)  10 (9.5)  1 (1.0) | 51 (40.5)  32 (25.4)  17 (13.5)  25 (19.8)  1 (0.8) | 80 (43.2)  57 (30.8)  25 (13.5)  21 (11.4)  2 (1.0) | 18 (40.9)  9 (20.5)  3 (6.8)  14 (31.8)  0 (0.0) |

^a^ Analysis by location excludes two respondents with unknown location. ^b^ Other areas include Gingie Village, Namoi Village, and out of town Walgett area. ^1^ Significant difference by sex at p<0.05. ^2^ Significant difference by age group at p<0.05. ^3^ Significant difference by location at p<0.05.

**Supplementary Table S7.** Water insecurity indicators Household Water InSecurity Experience experienced by participants during the last 12 months, overall, by sex, age and location (n, %)

| **QUESTIONS** | **OVERALL**  (*n*=251) | **BY SEX** | | **BY AGE GROUP** | | **BY LOCATION ^a^** | |
| --- | --- | --- | --- | --- | --- | --- | --- |
|  |  | **Males**  (*n*=112) | **Females**  (*n*=139) | **18 to 44 years**  (*n*=116) | **45 years and up** (*n*=135) | **Walgett town**  (*n*=203) | **Other areas ^b^**  (*n*=46) |
| **How often did you or anyone in your household worry that you would not have enough water for all of your needs?** | | | | | | | |
| Never  In one or two months  In some (3-6) months  In most (7-12) months  Don’t know | 82 (33.1)  38 (15.3)  40 (16.1)  88 (35.5)  0 (0.0) | 38 (34.6)  15 (13.6)  24 (21.8)  33 (30.0)  0 (0.0) | 44 (31.9)  23 (16.7)  16 (11.6)  55 (39.9) 0 (0.0) | 35 (31.0)  19 (16.8)  19 (16.8)  40 (35.4)  0 (0.0) | 47 (34.8)  19 (14.1)  21 (15.6)  48 (35.6)  0 (0.0) | 63 (31.3)  31 (15.4)  34 (16.9)  73 (36.3)  0 (0.0) | 17 (37.8)  7 (15.6)  6 (13.3)  15 (33.3)  0 (0.0) |
| **How often was your or your household's main water source interrupted or limited in any way (e.g. low or no water pressure, less water than expected, source dried up)? ^3^** | | | | | | | |
| Never  In one or two months  In some (3-6) months  In most (7-12) months  Don’t know | 84 (33.6)  71 (28.4)  56 (22.4)  37 (14.8)  2 (0.8) | 35 (31.5)  32 (28.8)  30 (27.0)  14 (12.6)  0 (0.0) | 49 (35.3)  39 (28.1)  26 (18.7)  23 (16.6)  2 (1.4) | 38 (33.0)  30 (26.1)  23 (20.0)  23 (20.0)  1 (0.9) | 46 (34.1)  41 (30.4)  33 (24.4)  14 (10.4)  1 (0.8) | 62 (30.7)  53 (26.2)  52 (25.7)  33 (16.3)  2 (1.0) | 21 (45.7)  18 (39.1)  4 (8.7)  3 (6.5)  0 (0.00) |
| **How often did you or anyone in your household NOT have enough water to drink as you would have liked?** | | | | | | | |
| Never  In one or two months  In some (3-6) months  In most (7-12) months  Don’t know | 94 (37.8)  40 (16.1)  48 (19.3)  66 (26.5)  1 (0.4) | 42 (37.8)  21 (18.9)  23 (20.7)  24 (21.6)  1 (0.9) | 52 (37.7)  19 (13.8)  25 (18.1)  42 (30.4)  0 (0.0) | 41 (36.0)  19 (16.7)  23 (20.2)  31 (27.2)  0 (0.0) | 53 (39.3)  21 (15.6)  25 (18.5)  35 (25.9)  1 (0.7) | 72 (35.9)  35 (17.4)  39 (19.4)  55 (27.7)  0 (0.0) | 21 (45.7)  5 (10.9)  9 (19.6)  11 (23.9)  0 (0.0) |
| **How often did you or anyone in your household change what you ate because there were problems with water (e.g. for washing foods, cooking)?** | | | | | | | |
| Never  In one or two months  In some (3-6) months  In most (7-12) months  Don’t know | 115 (46.2)  49 (19.7)  52 (20.9)  33 (13.3)  0 (0.0) | 57 (51.8)  20 (18.2)  21 (19.1)  12 (10.9)  0 (0.0) | 58 (41.7)  29 (20.9)  31 (22.3)  21 (15.1)  0 (0.0) | 48 (42.1)  28 (24.6)  26 (22.8)  12 (10.5)  0 (0.0) | 67 (49.6)  21 (15.6)  26 (19.3)  21 (15.6)  0 (0.0) | 88 (43.6)  39 (19.3)  45 (22.3)  30 (14.9)  0 (0.0) | 25 (55.6)  10 (22.2)  7 (15.6)  3 (6.7)  0 (0.0) |
| **How often have you or anyone in your household had to go without washing hands after dirty activities because of problems with water?** | | | | | | | |
| Never  In one or two months  In some (3-6) months  In most (7-12) months  Don’t know | 178 (71.5)  29 (11.7)  23 (9.2)  18 (7.2)  1 (0.4) | 83 (74.8)  15 (13.5)  9 (8.1)  4 (3.6)  0 (0.0) | 95 (68.8)  14 (10.1)  14 (10.1)  14 (10.1)  1 (0.7) | 81 (70.4)  17 (14.8)  12 (10.4)  5 (4.4)  0 (0.0) | 97 (72.4)  12 (9.0)  11 (8.2)  13 (9.7)  1 (0.8) | 142 (70.7)  23 (11.4)  22 (11.0)  14 (7.0)  0 (0.0) | 34 (73.9)  6 (13.0)  1 (2.2)  4 (8.7)  1 (2.2) |
| **How often have you or anyone in your household had to go without washing their body because of problems with water (e.g. not enough water, dirty, unsafe)?** | | | | | | | |
| Never  In one or two months  In some (3-6) months  In most (7-12) months  Don’t know | 160 (64.5)  44 (17.7)  23 (9.3) 20 (8.1)  1 (0.4) | 73 (65.8)  22 (19.8)  10 (9.0)  6 (5.4)  0 (0.0) | 88 (63.8)  22 (15.9)  13 (9.4)  14 (10.1)  1 (0.7) | 77 (67.0)  21 (18.3)  9 (7.9)  8 (7.0)  0 (0.0) | 84 (62.7)  23 (17.2)  14 (10.5)  12 (9.0)  1 (0.8) | 127 (63.2)  36 (17.9)  20 (10.0)  18 (9.0)  0 (0.0) | 32 (69.6)  8 (17.4)  3 (6.5)  2 (4.4)  1 (2.2) |
| **How often has problems with water meant that clothes could not be washed?** | | | | | | | |
| Never  In one or two months  In some (3-6) months  In most (7-12) months  Don’t know | 186 (79.5)  20 (8.6)  14 (6.0)  12 (5.1)  2 (0.9) | 79 (77.5)  11 (10.8)  6 (5.9)  4 (3.9)  2 (2.0) | 107 (81.1)  9 (6.8)  8 (6.1)  8 (6.1)  0 (0.0) | 91 (83.5)  7 (6.4)  7 (6.4)  3 (2.8)  1 (0.9) | 95 (76.0)  13 (10.4)  7 (5.6)  9 (7.2)  1 (0.8) | 147 (78.2)  17 (9.0)  14 (7.5)  8 (4.3)  2 (1.1) | 37 (84.1)  3 (6.8)  0 (0.0)  4 (9.1)  0 (0.0) |
| **How often have you or anyone in your household had to change schedules or plans due to problems with your water situation? (activities that may have been interrupted include caring for others, doing household chores, income-generating activities)** | | | | | | | |
| Never  In one or two months  In some (3-6) months  In most (7-12) months  Don’t know | 154 (62.4)  48 (19.4)  27 (10.9)  18 (7.3)  0 (0.0) | 71 (64.6)  23 (20.9)  12 (10.9)  4 (3.6)  0 (0.0) | 83 (60.6)  25 (18.3)  15 (11.0)  14 (10.2)  0 (0.0) | 71 (62.9)  22 (19.5)  14 (12.4)  6 (5.3)  0 (0.0) | 83 (62.0)  26 (19.4)  13 (9.7)  12 (9.0)  0 (0.0) | 120 (60.3)  38 (19.1)  27 (13.6)  14 (7.0)  0 (0.0) | 32 (69.6)  10 (21.7)  0 (0.0)  4 (8.7)  0 (0.0) |
| **How often did you or anyone in your household go to sleep thirsty because there was no drinkable water to drink?** | | | | | | | |
| Never  In one or two months  In some (3-6) months  In most (7-12) months  Don’t know | 158 (64.2)  34 (13.8)  26 (10.6)  27 (11.0)  1 (0.4) | 71 (65.1)  15 (13.8)  9 (8.3)  14 (12.9)  0 (0.0) | 87 (63.5)  19 (13.9)  17 (12.4)  13 (9.5)  1 (0.7) | 72 (63.7)  15 (13.3)  14 (12.4)  12 (10.6)  0 (0.0) | 86 (64.7)  19 (14.3)  12 (9.0)  15 (11.3)  1 (0.8) | 121 (61.1)  29 (14.7)  23 (11.6)  24 (12.1)  1 (0.5) | 35 (76.1)  5 (10.9)  3 (6.5)  3 (6.5)  0 (0.0) |
| **How often did you or anyone in your household have no usable or drinkable water whatsoever?** | | | | | | | |
| Never  In one or two months  In some (3-6) months  In most (7-12) months  Don’t know | 143 (57.9)  45 (18.2)  35 (14.2)  24 (9.7) 0 (0.0) | 64 (58.2)  22 (20.0)  14 (12.7)  10 (9.1)  0 (0.0) | 79 (57.7)  23 (16.8)  21 (15.3)  14 (10.2)  0 (0.0) | 62 (54.9)  20 (17.7)  21 (18.6)  10 (8.9)  0 (0.0) | 81 (60.5)  25 (18.7)  14 (10.5)  14 (10.5)  0 (0.0) | 112 (56.3)  36 (18.1)  30 (15.1)  21 (10.6)  0 (0.0) | 29 (63.0)  9 (19.6)  5 (10.9)  3 (6.5)  0 (0.0) |
| **How often did you or anyone in your household feel angry about your water situation?** | | | | | | | |
| Never  In one or two months  In some (3-6) months  In most (7-12) months  Don’t know | 78 (31.6)  31 (12.6)  23 (9.3)  115 (46.6)  0 (0.0) | 34 (30.9)  17 (15.5)  10 (9.1)  49 (44.6)  0 (0.0) | 44 (32.1)  14 (10.2)  13 (9.5)  66 (48.2)  0 (0.0) | 38 (33.6)  16 (14.2)  9 (8.0)  50 (44.3) 0 (0.0) | 40 (29.9)  15 (11.2)  14 (10.5)  65 (48.5)  0 (0.0) | 62 (31.2)  23 (11.6)  21 (10.6)  93 (46.7)  0 (0.0) | 15 (32.6)  8 (17.4)  2 (4.4)  21 (45.7)  0 (0.0) |
| **How often have problems with water caused you or anyone in your household to feel ashamed or excluded?** | | | | | | | |
| Never  In one or two months  In some (3-6) months  In most (7-12) months  Don’t know | 151 (60.9)  29 (11.7)  31 (12.5)  35 (14.1)  2 (0.8) | 68 (62.4)  10 (9.2)  17 (15.6)  13 (11.9)  1 (0.9) | 83 (59.7)  19 (13.7)  14 (10.1)  22 (15.8)  1 (0.7) | 71 (62.8)  14 (12.4)  13 (11.5)  14 (12.4)  1 (0.9) | 80 (59.3)  15 (11.1)  18 (13.3)  21 (15.6)  1 (0.8) | 119 (59.5)  24 (12.0)  28 (14.0)  27 (13.5)  2 (1.0) | 31 (67.4)  5 (10.9)  3 (6.5)  7 (15.2)  0 (0.0) |

^a^ Analysis by location excludes two respondents with unknown location. ^b^ Other areas include Gingie Village, Namoi Village, and out of town Walgett area

^1^ Significant difference by sex at p<0.05. ^2^ Significant difference by age group at p<0.05. ^3^ Significant difference by location at p<0.05.

**Supplementary Table S8.** Additional questions (developed through community consultation) to understand experiences of water insecurity during the last 12 months, overall, by sex, age and location (n, %)

| **QUESTIONS** | **OVERALL**  (*n*=251) | **BY SEX** | | **BY AGE GROUP** | | **BY LOCATION ^a^** | |
| --- | --- | --- | --- | --- | --- | --- | --- |
|  |  | **Males**  (*n*=112) | **Females**  (*n*=139) | **18 to 44 years**  (*n*=116) | **45 years and up**  (*n*=135) | **Walgett town**  (*n*=203) | **Other areas ^b^**  (*n*=46) |
| **What was your or your household's main water source? (select as many as relevant)** | | | | | | | |
| Tank water ^3^ | 77 (30.7) | 35 (31.3) | 42 (30.2) | 35 (30.2) | 42 (31.1) | 47 (23.2) | 29 (63.0) |
| Bore water | 72 (28.7) | 37 (33.0) | 35 (25.2) | 35 (30.2) | 37 (27.4) | 60 (29.6) | 12 (26.1) |
| Town water (supplied by council) | 199 (79.3) | 87 (77.7) | 112 (80.6) | 89 (76.7) | 110 (81.5) | 165 (81.3) | 32 (69.6) |
| Bottled water | 186 (74.1) | 79 (70.5) | 107 (77.0) | 91 (78.5) | 95 (70.4) | 152 (74.9) | 33 (71.7) |
| Source water or hydro-panel water ^3^ | 95 (37.9) | 42 (37.5) | 53 (38.1) | 40 (34.5) | 55 (40.7) | 68 (33.5) | 26 (56.5) |
| Other | 7 (2.8) | 2 (1.8) | 5 (3.6) | 5 (4.3) | 2 (1.5) | 7 (3.5) | 0 (0.0) |
| **How often did you or anyone in your household have to buy or rely on bottled water (donations or bought) because your main water source (tap/bore) was interrupted?** | | | | | | | |
| Never  In one or two months  In some (3-6) months  In most (7-12) months  Don’t know | 41 (16.5)  35 (14.1)  31 (12.5)  141 (56.6)  1 (0.4) | 18 (16.2)  18 (16.2)  16 (14.4)  58 (52.3)  1 (0.9) | 23 (16.7)  17 (12.3)  15 (10.9)  83 (60.1)  0 (0.0) | 18 (15.7)  14 (12.2)  19 (16.5)  64 (55.7)  0 (0.0) | 23 (17.2)  21 (15.7)  12 (9.0)  77 (57.5)  1 (0.8) | 32 (15.9)  26 (12.9)  21 (10.5)  121 (60.2)  1 (0.5) | 9 (19.6)  9 (19.6)  9 (19.6)  19 (41.3)  0 (0.0) |
| **How often did you or your household change methods of food preparation because there were problems with water (e.g., for washing foods, cooking)?** | | | | | | | |
| Never  In one or two months  In some (3-6) months  In most (7-12) months  Don’t know | 109 (44.0)  38 (15.3)  56 (22.6)  45 (18.2)  0 (0.0) | 53 (48.2)  16 (14.6)  23 (20.9)  18 (16.4)  0 (0.0) | 56 (40.6)  22 (16.0)  33 (23.9)  27 (19.6)  0 (0.0) | 44 (38.3)  21 (18.3)  27 (23.5)  23 (20.0)  0 (0.0) | 65 (48.9)  17 (12.8)  29 (21.8)  22 (16.5)  0 (0.0) | 83 (41.5)  29 (14.5)  48 (24.0)  40 (20.0)  0 (0.0) | 24 (52.2)  9 (19.6)  8 (17.4)  5 (10.9)  0 (0.0) |
| **How often were you concerned about the quality (taste, smell, contamination) of your main water source?** | | | | | | | |
| Never  In one or two months  In some (3-6) months  In most (7-12) months  Don’t know | 22 (9.0)  31 (12.7)  39 (15.9)  152 (62.0)  1 (0.4) | 8 (7.3)  16 (14.7)  16 (14.7)  68 (62.4)  1 (0.9) | 14 (10.3)  15 (11.0)  23 (16.9)  84 (61.8)  0 (0.0) | 12 (10.7)  17 (15.2)  15 (13.4)  68 (60.7)  0 (0.0) | 10 (7.5)  14 (10.5)  24 (18.1)  84 (63.2)  1 (0.8) | 17 (8.6)  22 (11.2)  31 (15.7)  126 (64.0)  1 (0.5) | 5 (10.9)  9 (19.6)  7 (15.2)  25 (54.4)  0 (0.0) |
| **How often did you have to buy or rely on donations of bottled water because you were worried about the quality (taste/smell/contamination) of the main water source?** | | | | | | | |
| Never  In one or two months  In some (3-6) months  In most (7-12) months  Don’t know | 34 (13.7)  28 (11.2)  32 (12.9)  154 (61.9)  1 (0.4) | 13 (11.8)  16 (14.6)  17 (15.5)  63 (57.3)  1 (0.9) | 21 (15.1)  12 (8.6)  15 (10.8)  91 (65.5)  0 (0.0) | 17 (14.9)  14 (12.3)  13 (11.4)  70 (61.4)  0 (0.0) | 17 (12.6)  14 (10.4)  19 (14.1)  84 (62.2)  1 (0.7) | 28 (13.9)  22 (11.0)  23 (11.4)  128 (63.7)  0 (0.0) | 6 (13.0)  6 (13.0)  8 (17.4)  26 (56.5)  0 (0.0) |
| **How often did you NOT have enough water for indoor use such as cooking, bathing, washing and cleaning as you would have liked?** | | | | | | | |
| Never  In one or two months  In some (3-6) months  In most (7-12) months  Don’t know | 122 (49.0)  39 (15.7)  48 (19.3)  38 (15.3)  2 (0.8) | 59 (53.6)  18 (16.4)  20 (18.2)  13 (11.8)  0 (0.0) | 63 (45.3)  21 (15.1)  28 (20.1)  25 (18.0)  2 (1.4) | 55 (48.3)  18 (15.8)  25 (21.9)  15 (13.2)  1 (0.9) | 67 (49.6)  21 (15.6)  23 (17.0)  23 (17.0)  1 (0.7) | 94 (46.8)  32 (15.9)  40 (19.9)  33 (16.4)  2 (1.0) | 26 (56.5)  7 (15.2)  8 (17.4)  5 (10.9)  0 (0.0) |
| **How often did you NOT have enough water for outdoor use (gardening) as you would have liked?** | | | | | | | |
| Never  In one or two months  In some (3-6) months  In most (7-12) months  Don’t know | 149 (62.1)  28 (11.7)  28 (11.7)  32 (13.3)  3 (1.3) | 69 (64.5)  10 (9.4)  11 (10.3)  15 (14.0)  2 (1.9) | 80 (60.2)  18 (13.5)  17 (12.8)  17 (12.8)  1 (0.8) | 73 (67.0)  14 (12.8)  10 (9.2)  12 (11.0)  0 (0.0) | 76 (58.0)  14 (10.7)  18 (13.7)  20 (15.3)  3 (2.3) | 116 (60.4)  22 (11.5)  27 (14.1)  25 (13.0)  2 (1.0) | 32 (69.6)  6 (13.0)  1 (2.2)  6 (13.0)  1 (2.2) |

^a^ Analysis by location excludes two respondents with unknown location. ^b^ Other areas include Gingie Village, Namoi Village, and out of town Walgett area

^1^ Significant difference by sex at p<0.05. ^2^ Significant difference by age group at p<0.05. ^3^ Significant difference by location at p<0.05.
